# The Associations between Self-Determined Motivation, Multidimensional Self-Efficacy, and Device-Measured Physical Activity

**DOI:** 10.3390/ijerph18158002

**Published:** 2021-07-29

**Authors:** I-Hua Chu, Yu-Ling Chen, Pei-Tzu Wu, Wen-Lan Wu, Lan-Yuen Guo

**Affiliations:** 1 Department of Sports Medicine, Kaohsiung Medical University, Kaohsiung 807, Taiwan; ylchen60@gm.ntus.edu.tw (Y.-L.C.); wenlanwu@kmu.edu.tw (W.-L.W.); yuen@kmu.edu.tw (L.-Y.G.); 2 Ph.D. Program in Biomedical Engineering, College of Medicine, Kaohsiung Medical University, Kaohsiung 807, Taiwan; 3 Department of Medical Research, Kaohsiung Medical University Hospital, Kaohsiung 807, Taiwan; 4 School of Physical Therapy & Athletic Training, Pacific University, Hillsboro, OR 97123, USA; eliza.wu@pacificu.edu

**Keywords:** self-determined motivation, self-efficacy, accelerometer, physical activity, exercise behavior, adults

## Abstract

Self-determined motivation (SDT) and self-efficacy theory have been widely used for understanding individuals’ physical activity motivation and self-efficacy. However, there is a gap of evidence on the relations between SDT and multidimensional self-efficacy with device-measured physical activity in healthy adults. Questionnaires including the behavior regulation in exercise questionnaire version 2 (BREQ-2) and the multidimensional self-efficacy for exercise scale (MSES) were completed by the participants at baseline. All participants wore an accelerometer for seven days to record their physical activities at baseline and eight-week follow up. In total, thirty healthy adults completed the study (12 men, 18 women). The results showed that intrinsic motivation and scheduling self-efficacy had significantly positive associations with moderate-to-vigorous physical activity energy expenditure and duration. Multiple regression analysis showed that the relative autonomy index, task and scheduling efficacy could predict physical activity at baseline, but no SDT or self-efficacy variable could predict physical activity behavior after eight weeks. These results showed that the associations between motivation and self-efficacy with physical activity might change within a short period of time, which suggests that the regular assessment of motivation and self-efficacy might be needed in interventional programs to promote continued physical activity participation in healthy adults.

## 1. Introduction

Physical inactivity has been found to be associated with several non-communicable diseases such as coronary heart disease, type 2 diabetes, breast cancer, and colon cancer worldwide [1]. The recent evidence also showed that participating in a higher level of physical activity was associated with a reduced risk of premature mortality in adults [2]. In addition, lower to moderate intensity of physical activity was found to help reduce the symptom of depression in women [3]. Based on the existing scientific evidence, the World Health Organization (WHO) recently updated its guidelines on physical activity and sedentary behavior, and recommended adults aged 18–64 to participate at least 150 min of moderate-to-vigorous physical activity (MVPA) per week in order to achieve health benefits [4]. However, the prevalence of physical inactivity remains high in the worldwide population, with 23% of adults not meeting the WHO physical activity guidelines, and the percentage could rise to as high as 80% in some countries [5]. While physical inactivity is garnering more attention as a global public health issue, an increasing amount of physical activity promotion interventions are being designed, and researchers are encouraged to reconsider how they incorporate the behavior change theories into their physical activity and health intervention [6]. Among all the behavior change theories, the self-determination theory [7] and self-efficacy theory [8] are two of the reputable theories that have been applied to explain or predict behavioral change (e.g., the initiation of regular exercise) [9,10]. As these two theories are both based on the ideology that humans are agents of their actions, it was encouraged that more studies integrate these two motivational theories in physical activity [9].

Self-determination theory (SDT) [7] is a theory that emphasizes the human motivation based on various degrees of autonomy with multiple constructs. In SDT, autonomy refers to behaviors being self-determined or freely initiated by the individual, and multiple constructs comprise intrinsic motivation, extrinsic motivation, and amotivation as the motivational continuum. Previous studies found that SDT could predict physical activity after 1-month follow up [11]. Evidence also demonstrated that the SDT explained more than 30% of the variance in physical activity among adult women [3]. The self-efficacy theory describes self-efficacy as one’s beliefs which are important and are the primary determinant of physical activity. The evidence shows that self-efficacy is positively associated with physical activity across age groups [12,13,14], and it could be a sensitive factor to predict the physical activity level of older adults [15]. Self-efficacy for exercise/physical activity can be characterized into three domains, including task, scheduling, and coping self-efficacy, and it was found that this multidimensional structure of self-efficacy was correlated and influenced the individuals’ physical activity [16].

Although SDT and multidimensional self-efficacy have been shown to be determinants or predictors of physical activity behavior, most of the previous studies assessed physical activity levels using self-reported physical activity questionnaires. In spite of the good reliability and validity for self-reported physical activity measures, such as the 7-day physical activity recall [17], self-reports of physical activity are likely to be influenced by social desirability and recall bias [18,19]. Therefore, device-based physical activity measures are increasingly used in the research of physical activity. However, only a few studies explored the association between the SDT and the accelerometer-determined physical activity [20,21,22], and most of these studies were cross-sectional in their design. Even though evidence on the association of self-efficacy and device-measured physical activity is emerging, there is lack of evidence on the association of multidimensional self-efficacy with physical activity. Overall, little evidence is based on studies that prospectively examine the relationship between the SDT and multidimensional self-efficacy with device-measured physical activity in adults. Therefore, the purpose of this study was to: (1) examine the relationship between self-determined motivation and self-efficacy and device-measured physical activity in adults, and (2) investigate whether self-determined motivation and self-efficacy variables would be sensitive predictors of future physical activity behavior in healthy adults. We hypothesized that (1) self-determined motivation and self-efficacy variables would be positively associated with physical activity level, and (2) self-determined motivation and self-efficacy variables would be able to predict physical activity levels in 8 weeks.

## 2. Methods

### 2.1. Participants

Participants were initially recruited via posters in the community around the Kaohsiung Medical University campus, and other approaches such as emails and word of mouth were also applied throughout the recruitment period. The criteria for enrollment included: (1) participants had to be between 20 and 65 years of age, and (2) have a body mass index (BMI) between 18.5 and 27 kg/m^2^. Participants were excluded if they (1) had physical contraindications to participate in physical activity, (2) engaged in any water sports activities (due to the limitation of the accelerometer), or (3) had any cardiovascular or other disease. The study protocol was approved by the Kaohsiung Medical University Chung-Ho Memorial Hospital Institutional Review Board (KMUH-IRB-20110072). All participants were informed of the benefits and risks of the investigation, and written informed consent was received from all participants.

### 2.2. Procedures

During the first visit, participants were asked to complete a series of questionnaires, including the Behavioral Regulation in Exercise Questionnaire-2 (BREQ-2) [23], and the multidimensional self-efficacy for exercise scale (MSES) [24]. The participants were also given an accelerometer and instructed to wear the device for seven days (Week 0). Eight weeks later, participants wore the accelerometer again when they returned for the follow-up measurement (Week 9).

### 2.3. Measures

#### 2.3.1. Self-Determined Motivation

Participants’ self-determined motivation for exercising was measured with the BREQ-2. The BREQ-2 comprises five subscales, a total of 19 items, that assess intrinsic motivation (4 items), identified regulation (4 items), introjected regulation (3 items), external regulation (4 items), and amotivation (4 items). Example items are: “I exercise because it’s fun” (intrinsic motivation); “I value the benefits of exercise” (identified regulation); “I feel guilty when I don’t exercise” (introjected regulation); “I exercise because other people say I should” (external regulation); and “I don’t see why I should have to exercise” (amotivation). Participants responded to each item on a 5-point Likert-type scale ranging from 0 = “not true for me” to 4 = “very true for me.” A previous study has shown acceptable internal consistencies (Cronbach’s alpha) of the subscales ranging from 0.72 (identified regulation) to 0.83 (intrinsic regulation) [23]. Furthermore, the relative autonomy index (RAI), a single score derived from the subscales, was calculated to present the degree to which respondents feel self-determined. Each subscale of regulation was weighted (i.e., amotivation = −3, external regulation = −2, introjection = −1, identification = +2, and intrinsic regulation = +3), and then these weighted scores were summed as the RAI. The traditional Chinese version of the BREQ-2 was translated prior to the current study. The test of the reliability and validity of the translated version was conducted in one hundred university students, who completed the translated BREQ-2 and repeated the same questionnaire after two weeks. The Cronbach’s α for each subscale of the translated BREQ-2 ranged from 0.72 to 0.94, suggesting good internal consistency. Intraclass correlation coefficients ranged from 0.73 to 0.89, suggesting acceptable test–retest reliability.

#### 2.3.2. Self-Efficacy Assessment 

Participant’s self-efficacy for exercise was evaluated using MSES [24], which consists of three dimensions: task efficacy (i.e., complete your exercise using the proper technique, follow directions to complete exercise, perform all of the required movements), coping efficacy (i.e., exercise when you feel discomfort, exercise when you lack energy, exercise when you do not feel well) and scheduling efficacy (i.e., include exercise in your daily routine, consistently exercise 3 times/week, arrange your schedule to include regular exercise). All participants were asked to assess the confidence level in maintaining exercise behaviors for the following 6 months, and to answer these questions on 10-point confidence scales ranging from 0 = not confident at all to 10 = completely confident. The final score for self-efficacy was calculated by averaging the scores of these 3 dimensions. The internal consistencies (Cronbach’s alpha) were 0.81 for both task efficacy and coping efficacy, and 0.91 for scheduling efficacy [16]. The traditional Chinese version of the MSES was translated prior to the current study. The test of the reliability and validity of the translated version was conducted in the same way at the same time as BREQ-2, as described in the previous section. The Cronbach’s α for each subscale of the translated MSES ranged from 0.79 to 0.94, suggesting good internal consistency. Intraclass correlation coefficients ranged from 0.82 to 0.89, suggesting acceptable test–retest reliability.

#### 2.3.3. Physical Activity Behavior 

Each participant was given a 3-axis accelerometer (ActiGraph GT3X, Pensacola, FL, USA) to wear on their waist to record their physical activity. The collected data were then processed using the ActiLife 5 software (ActiGraph, Pensacola, FL, USA). The cut points that were used in the current study were a combination of Freedson’s [25] and Matthews’s [26] cut points. Sedentary behavior and physical activity were categorized by counts per minute (CPM) as: sedentary (0–99 cpm), light (100–759 cpm), lifestyle (760–1951 cpm), moderate (1952–5724 cpm) and vigorous (>5724 cpm). The total time (duration; minutes) and energy expenditure (kcal) of the recorded moderate-to-vigorous-intensity physical activity (MVPA) over the 7-day period were calculated and analyzed using the ActiLife 5 software.

### 2.4. Statistical Analyses

Descriptive results were expressed in mean ± standard deviation (SD) values. The outcome variables were the two physical activity behaviors (i.e., energy expenditure and duration of MVPA), and the independent variables were the results from the BREQ-2 and MSES questionnaire. Continuous data were checked for normality using Kolmogorov–Smirnov tests. Pearson’s correlation for normally distributed data or Spearman’s correlation for non-normally distributed data were performed to test univariate relationships between the outcome and independent variables. Simultaneous multiple regression analyses were conducted to examine the relationships between the outcome variable with the independent variables at baseline and follow up. At baseline, gender and age were controlled and added to the multiple regression model. At follow up, gender, age, and physical activity at baseline were controlled and added to the multiple regression model. All statistical analyses were completed using the Statistical Package for the Social Sciences (SPSS Version 25.0; SPSS Inc., Chicago, IL, USA). Type I error was set at α = 0.05 (two-tailed) for all tests.

## 3. Results

### 3.1. Participant’s Characteristics

A total of 38 participants were recruited, and 30 of them (12 men; mean age 33.8 ± 12.0 years) completed the study. The mean height, body weight, and BMI were 165.3 ± 9.7 cm, 66.3 ± 11.3 kg, and 24.1 ± 3.0 kg/m^2^, respectively. The results of the self-determined motivation and self-efficacy at baseline and physical activity at baseline (week 0) and follow up (week 9) are presented in Table 1. The mean scores were higher in intrinsic motivation and identified regulation as compared to the other three subscales. Amotivation received the lowest score. In terms of self-efficacy, task efficacy had the highest mean score, followed by scheduling efficacy and coping efficacy.

### 3.2. The Association of SDT and MSES with Physical Activity

At baseline, intrinsic motivation was positively and significantly correlated with MVPA energy expenditure (ρ = 0.36, *p* < 0.05) and duration (ρ = 0.55, *p* < 0.01). RAI was positively and significantly correlated with the MVPA duration (ρ = 0.47, *p* < 0.05). No significant correlation was observed between other motivation subscales with the physical activity behavior. Scheduling efficacy was positively and significantly correlated with MVPA energy expenditure (ρ = 0.45, *p* < 0.05) and duration (ρ = 0.48, *p* < 0.05), while no significant correlation was observed in task efficacy or coping efficacy with the physical activity behavior. At follow up, intrinsic motivation was the only motivation variable that positively and significantly correlated with MVPA energy expenditure (ρ = 0.42, *p* < 0.05). Scheduling efficacy was the only self-efficacy variable that positively and significantly correlated with MVPA energy expenditure (ρ = 0.44, *p* < 0.05). The detailed information of the association between the SDT and MSES variable with MVPA behavior can be found in Supplementary Material (Tables S1–S4).

### 3.3. The SDT and MSES Predictors of MVPA at Baseline

The results of the regression analysis showed that none of the subscale of SDT was a significant predictor of MVPA energy expenditure and duration at the baseline assessment (Table 2). On the other hand, when age and gender were controlled, the regression model showed that RAI could predict MVPA duration and significantly explained 18% of the variance in MVPA duration. When age and gender were controlled, together, the multidimensional self-efficacy significantly explained 58% and 45% of the variance in the energy expenditure and duration of MVPA, respectively. The model showed that the task efficacy and scheduling efficacy were significant predictors of the energy expenditure and duration of MVPA.

### 3.4. The SDT and MSES predictors of MVPA at Follow Up

At follow up, when age, gender and baseline MVPA were controlled, none of the subscale of SDT or RAI was a significant predictor of MVPA energy expenditure and duration (Table 3). Similarly, none of the multidimensional self-efficacy was a significant predictor of MVPA energy expenditure and duration.

## 4. Discussion

The present prospective study examined the relationship between SDT and multidimensional self-efficacy theory and device-measured physical activity in adults. It also investigated the roles of SDT and multidimensional self-efficacy in the prediction of future physical activity behavior in healthy adults. In general, the findings of this study demonstrated that, when the device-based physical activity measurement was applied, SDT and multidimensional self-efficacy may be associated with MVPA energy expenditure and duration. It was also found that RAI and scheduling self-efficacy could predict the energy expenditure and duration of MVPA.

### 4.1. The Relationship between Self-Determined Motivation and Physical Activity

This present study found that intrinsic motivation was the only SDT variable that significantly correlated with physical activity behavior. The results revealed consistent significant and positive associations of the intrinsic motivation with MVPA energy expenditure at both baseline and follow up. The positive and significant correlation between intrinsic motivation with MVPA duration was only found at baseline. This finding is in line with the results found in the study conducted by Sebire and colleagues, which used the baseline data of a randomized controlled trial for examining the associations between physical activity motivation and device (ActiGraph)-measured physical activity among children. It found that intrinsic motivation was the only physical activity behavior motivation that had a significant positive association with MVPA in children [27]. However, this finding is different to the recent cross-sectional study conducted by Tao and colleagues. Their study also used the wrist-worn ActiGraph to assess the participating college students’ physical activity and found no significant correlation between autonomous motivations (i.e., intrinsic and identified regulation) and the duration of MVPA [21]. Notably, in Tao’s study, the participants were less active (1.57 min of MVPA/day) compared to the relatively more active participants in this current study (24.98 min of MVPA/day) and Sebire’s study (59 min of MVPA/day) [28]. The difference in physical activity level of the participants could potentially explain the inconsistency in the evidence of the association between the autonomous motivations and MVPA.

The current study found no significant correlation between intrinsic motivation at baseline with the duration of MVPA at follow up. RAI was the only predictor of MVPA duration at baseline, and no SDT variable at baseline could significantly predict MVPA behavior at follow up. This result is contradicted to the evidence found in the previous prospective studies. For example, Standage and colleagues assessed moderate intensity exercise using a combined accelerometer and heart rate monitor in college students. The results of their study showed that both intrinsic motivation and identified regulation were significantly correlated with the duration of moderate intensity exercise [20]. They also found that autonomous motivation (average score of intrinsic motivation and identified regulation subscales) could be a significant predictor of moderate intensity exercise. Another longitudinal study conducted by Sebire and colleagues, which also used ActiGraph to assess physical activity, also found a significant and positive correlation between autonomous motivation (i.e., intrinsic and identified regulation) and the MVPA duration in healthy adults [28]. Their results also showed that the autonomous motivation could predict and significantly explain 15% of the variance in MVPA duration. The possible reason of the inconsistent results could be due to the difference in the length of the observational period. In both Standage’s and Sebire’s studies, the physical activity level of their participants was measured one week after the BREQ was completed. In the present study, the physical activity was followed for a longer period of time (eight weeks). Therefore, more research that employs a longer observational period and multiple time points of device-based physical activity measurement is needed to further examine the association between intrinsic motivation and identified regulation with device-measured physical activity.

There are similar and different results when comparing the findings of the current study to the previous studies that measured physical activity using a self-report questionnaire. Similarly, a significant correlation between intrinsic motivation with the duration of MVPA was also found in a population with different conditions such as obesity and cardiovascular disease [29,30,31,32]. However, the finding of no significant correlation between extrinsic motivation (i.e., external, introjected and identified regulations) with the studied device-measured MVPA variables were different from some of the existing evidence which was based on the self-report measurement. For example, the cross-sectional study conducted by Duncan and colleagues found that introjected and identified regulations were significantly and positively correlated with the self-report physical activity in a regular exerciser. The randomize controlled trail (RCT) conducted by Silva and colleagues also found that the SDT-based intervention improved participants’ introjected and identified regulation, which led to increased levels of physical activity at the 12-month follow-up [32]. Teixeira and colleagues summarized the existing evidence and suggested that the controlled types of motivation showed negative or null association with exercise behavior [33]. Therefore, although the findings of the present study were aligned with the tenet of SDT, which suggests that introjected regulation lacks a high degree of self-autonomy and could have relatively less influence on physical activity, further evidence is needed to explore the relationship between the extrinsic motivation with device-based physical activity among different groups of a population.

Studies that mainly prospectively examined the relationships between exercise motivation and self-report physical activity suggested that overall, self-determined motivation could predict and account for 11 to 12% of the variance in physical activity in participants with cardiovascular conditions [29,30,31]. It was also suggested in Teixeria and colleagues’ systematic review that positive association was more consistently found between identified regulation and exercise behavior in multivariate analyses [33]. As the majority of the studies included in Teixeria and colleagues’ systematic review applied self-report measurement of physical activity, the desirability bias of the self-report method [34] could potentially be the reason of the contradiction in evidence. Our findings urge further research that examines whether the association between SDT and physical activity behavior would be different when physical activity is measured via self-report versus a device.

### 4.2. The Relationship between Self-Efficacy and Physical Activity

Based on the evidence from mostly cross-sectional or experimental studies, self-efficacy is consistently positively correlated with physical activity in adults [12]. In the previous studies, scheduling efficacy is the aspect of self-efficacy that most closely linked to self-report physical activity behavior [35,36]. Moreover, compared to other types of self-efficacy variables, scheduling self-efficacy was most strongly related to exercise adherence in participants undergoing post-operational cardiac rehabilitation, which involved structured exercise programs [36]. This implied that regardless of declined physical ability to perform exercise, scheduling efficacy remains an important factor of physical activity behavior. Generally, the findings from the current prospective study support the existing evidence and provide a further interrogation of data by applying the multidimensional self-efficacy. The current study found that only scheduling self-efficacy was consistently and significantly correlated with the device-measured MVPA energy expenditure at baseline and follow up. However, the positive association between scheduling self-efficacy and MVPA duration was only found at baseline. Such findings are different from a study conducted by McAuley and colleagues, which found a significant positive correlation between scheduling efficacy and long-term physical activity behavior [37]. As McAuley and colleagues measured the participants’ exercise behavior by asking the frequency of exercise through a telephone interview, the different methods in physical activity measurement could possibly relate to the different findings in the current study to the existing evidence. Similarly, scheduling and task self-efficacy were the predictors of the MVPA energy expenditure and duration at baseline but not at follow up. Prior studies have observed that, contrary to individuals who have physical limitations to perform exercise, task efficacy did not significantly affect long-term and consistent physical activity behavior in healthy individuals. In addition, task efficacy was shown to be more related to behavioral intention than behavior [38], which is aligned with the findings in the present study that task efficacy was not associated with the energy expenditure nor the duration of MVPA. More evidence is needed to understand the association of multidimensional self-efficacy with long-term device-measured physical activity.

### 4.3. Applications and Limitations

The results of the current study suggested that physical activity interventions may consider including programs that target enhancing individuals’ intrinsic motivation and scheduling efficacy to increase the likelihood of physical activity participation in a healthy population. However, the limitations of this present study include: (1) small sample size; it serves as a pilot study, and further research with a larger sample size is needed to provide more robust and powered results; (2) this eight-week observational study only provides preliminary evidence on the relatively short-term association and prediction of SDT and SET with physical activity behavior. However, our findings suggested that the association of SDT and SET variables with physical activity behavior could change within a short period of time. Future studies with longer observational periods and multiple measurement points are needed to further examine the associations of SDT and SET with long-term device-measured physical activity; (3) variations in physical activity habits and lifestyles due to various socioeconomic factors were not taken into account. Future research focusing on the relationships between other moderating factors (e.g., health status, occupation, education level, stage of physical activity) and exercise motivation in specific populations are needed.

## 5. Conclusions

Some aspects of self-determination theory and self-efficacy theory were associated with 8-week physical activity level. However, none of them were a significant predictor of physical activity behaviors, including energy expenditure and duration, using device-based physical activity assessment over eight weeks of observation. Of all the examined motivation and self-efficacy variables, intrinsic motivation and scheduling efficacy showed the most potential to consistently relate or influence physical activity behavior in healthy adults. The associations between the individual’s motivation and self-efficacy with physical activity might change within a short period of time. Therefore, long-term physical activity intervention should consider regularly monitoring the possible changes in the individual’s motivation and self-efficacy of physical activity in order to provide more efficient support to facilitate regular physical activity habits or improve exercise adherence.

## Figures and Tables

**Table 1:** Descriptive results of self-determined motivation, self-efficacy, and physical activity behavior.

Variables	Mean	SD
Self-determined motivation scores		
Amotivation	0.43	0.60
Extrinsic regulation	0.92	0.57
Introjected regulation	2.02	1.12
Identified regulation	2.85	0.76
Intrinsic motivation	2.97	0.71
Self-efficacy scores		
Task efficacy	5.93	2.36
Scheduling efficacy	5.89	2.74
Coping efficacy	2.61	2.07
Physical activity at baseline		
MVPA energy expenditure (kcal/week)	839.17	678.03
MVPA duration (minutes/week)	174.87	118.40
Physical activity at follow up		
MVPA energy expenditure (kcal/week)	494.55	371.54
MVPA duration (minutes/week)	102.82	70.633

Note: MVPA = moderate-to-vigorous physical activity.

**Table 2:** Predictors of MVPA energy expenditure and duration at baseline (Week 0).

	Energy Expenditure				Duration			
**Model 1**	**F**	**df**	**R^2^adj**	**β**	**F**	**df**	**R^2^adj**	**β**
	2.05	7	0.20		1.47	7	0.10	
Age				−0.21				−0.07
Gender				−0.43 *				−0.15
Amotivation				0.15				0.14
External regulation				−0.04				−0.06
Introjected regulation				−0.32				−0.12
Identified regulation				0.45				0.17
Intrinsic motivation				0.14				0.45
**Model 2**	**F**	**df**	**R^2^adj**	**β**	**F**	**df**	**R^2^adj**	**β**
	4.60 *	3	0.27		3.07 *	3	0.18	
Age				−0.03				−0.08
Gender				−0.49 **				−0.27
RAI				0.23				0.37 *
**Model 3**	**F**	**df**	**R^2^adj**	**β**	**F**	**df**	**R^2^adj**	**β**
	8.92 ***	5	0.58		5.70 **	5	0.45	
Age				−0.35 *				−0.41 *
Gender				−0.47 **				−0.24
Task efficacy				−1.23 ***				−1.23 **
Coping efficacy				0.12				0.07
Scheduling efficacy				1.25 ***				1.39 ***

Note: * *p* < 0.05, ** *p* < 0.01, *** *p* < 0.001. MVPA = moderate-to-vigorous physical activity; RAI = relative autonomy index.

**Table 3:** Predictors of MVPA energy expenditure and duration at follow up (Week 9).

	Energy Expenditure				Duration			
**Model 1**	**F**	**df**	**R^2^adj**	**β**	**F**	**df**	**R^2^adj**	**β**
	1.74	8	0.18		1.09	8	0.03	
Age				0.04				−0.02
Gender				−0.33				−0.21
Baseline MVPA				0.13 ^a^				0.06 ^b^
Amotivation				0.39				0.55
External regulation				−0.23				−0.57
Introjected regulation				0.12				0.08
Identified regulation				0.07				0.13
Intrinsic motivation				0.21				0.04
**Model 2**	**F**	**df**	**R^2^adj**	**β**	**F**	**df**	**R^2^adj**	**β**
	3.12 *	4	0.24		1.09	4	0.01	
Age				−0.01				0.01
Gender				−0.48 *				−0.34
Baseline MVPA				0.16 ^a^				0.10 ^b^
RAI				0.04				0.03
**Model 3**	**F**	**df**	**R^2^adj**	**β**	**F**	**df**	**R^2^adj**	**β**
	3.15 *	6	0.32		0.88	6	−0.03	
Age				−0.24				−0.02
Gender				−0.67 *				−0.27
Baseline MVPA				−0.20 ^a^				0.01 ^b^
Task efficacy				−0.90				−0.09
Coping efficacy				−0.08				−0.24
Scheduling efficacy				0.91				0.34

Note: * *p* < 0.05. MVPA = moderate-to-vigorous physical activity; RAI = relative autonomy index; a = controlled for baseline MVPA energy expenditure, b = controlled for baseline MVPA duration.

## Data Availability

Data can be made available upon request.

## Supplementary Materials

The following are available online at https://www.mdpi.com/article/10.3390/ijerph18158002/s1, Table S1: Correlations between Self-determined Motivation and Physical Activity at Baseline, Table S2: Correlations between Self-efficacy and Physical Activity at Baseline, Table S3: Correlations between Self-determined Motivation and Physical Activity at Follow-up, Table S4: Correlations between Self-efficacy and Physical Activity at Follow-up.
