# Supplementary material for: The Associations between Self-Determined Motivation, Multidimensional Self-Efficacy, and Device-Measured Physical Activity
Source: Int J Environ Res Public Health. 2021 Jul 29;18(15):8002. doi: 10.3390/ijerph18158002 (PMC8345365; doi:10.3390/ijerph18158002)
Supplement: Supplementary file 1 [file ijerph-18-08002-s001.zip › ijerph-1262410-supplementary.pdf]

Table S1. Correlations between Self-determined Motivation and Physical Activity at Baseline.

Variable	1	2	3	4	5	6	7	8
1. Amotivation	-							
2. External regulation	0.42*	-						
3. Introjected regulation	-0.23	0.12	-					
4. Identified regulation	-0.39**	-0.02	0.65**	-				
5. Intrinsic motivation	-0.20**	-0.39*	0.38	0.57**	-			
6. RAI	-0.56**	-0.64**	0.20	0.58**	0.82**	-		
7. MVPA energy expenditure (kcal/week)	-0.16	-0.15	0.10	0.25	0.36*	0.33	-	
8. MVPA duration (min/week)	0.05	-0.31	0.18	0.28	0.55**	0.47*	0.92**	-

Note：*p<0.05, **p<0.01. MVPA= moderate to vigorous physical activity; RAI =relative autonomy index

Table S2. Correlations between Self-efficacy and Physical Activity at Baseline.

Variable	1	2	3	4	5
1. Task efficacy	-				
2. Coping efficacy	0.62**	-			
3. Scheduling efficacy	0.76**	0.55**	-		
4. MVPA energy expenditure (kcal/kg/week)	0.14	0.26	0.45*	-	
5. MVPA duration (min/week)	0.20	0.18	0.48*	0.92**	-

Note：*p<0.05, **p<0.01. MVPA = moderate to vigorous physical activity.

Table S3. Correlations between Self-determined Motivation and Physical Activity at Follow-up.

Variable	1	2	3	4	5	6	7	8
1. Amotivation	-							
2. External regulation	0.42**	-						
3. Introjected regulation	-0.23	0.12	-					
4. Identified regulation	-0.39*	0.02	0.65**	-				
5. Intrinsic motivation	-0.20	-0.39**	0.38**	0.57**	-			
6. RAI	-0.56**	-0.64**	0.20	0.58**	0.82**	-		
7. MVPA energy expenditure (kcal/week)	0.17	-0.19	0.16	0.24	0.42*	0.31	-	
8. MVPA duration (min/week)	0.19	-0.18	0.05	0.11	0.24	0.2	0.96**	-

Note：*p<0.05, **p<0.01. MVPA= moderate to vigorous physical activity; RAI =relative autonomy index

Table S4. Correlations between Self-efficacy and Physical Activity at Follow-up.

Variable	1	2	3	4	5
1. Task efficacy	-				
2. Coping efficacy	0.62**	-			
3. Scheduling efficacy	0.76**	0.55**	-		
4. MVPA energy expenditure (kcal/kg/week)	0.16	-0.08	0.44*	-	
5. MVPA duration (min/week)	0.13	-0.17	0.33	0.96**	-

Note：*p<0.05, **p<0.01. MVPA = moderate to vigorous physical activity.
